# Disrespect and abuse in maternity care in a low-resource setting in Tanzania: Provider’s perspectives of practice

**DOI:** 10.1371/journal.pone.0281349

**Published:** 2023-03-22

**Authors:** Loveluck K. Mwasha, Lucy M. Kisaka, Eunice S. Pallangyo

**Affiliations:** The Aga Khan University- School of Nursing and Midwifery, Dar es Salaam, Tanzania; Public Health Foundation of India, INDIA

## Abstract

**Background:**

Globally, women experience disrespectful and abusive care from maternity healthcare providers at health facilities, committed intentionally or unintentionally, particularly during labor and delivery. Disrespectful care affects women’s childbirth experience and birth outcomes.

**Methods:**

This study used a descriptive qualitative design to obtain thick and rich data on disrespect and abuse in maternity care in a low-resource setting in Tanzania. Three days workshop was conducted at the Aga Khan University comprising maternity healthcare providers from diverse settings. The workshop was designed based on the existing evidence and anecdotal data and inspired by the authors’ experiences of disrespectful and abusive care (stereotyping clients, not listening to client’s/relatives’ concerns, unconsented care) as a client, relative, or observant of colleagues. The targeted audience was maternity healthcare providers from public and private health facilities in the Dar es Salaam region. Data collection encompassed individual responses (reflection of practice) obtained by individuals, anonymously written reflections of practice, and compiled notes from group discussions. Data were analysed thematically guided by six steps described by Braun and Clerk.

**Results:**

A total of 80 maternity healthcare providers participated in the workshop from various health facilities, including dispensaries (*n* = 25), health centres (*n* = 2), and hospitals (*n* = 3) located in semi-urban Dar es Salaam.

Four main themes were identified from the data: Physical and verbal abuse; Lack of professional ethics and integrity; Vulnerable working environment; Abuse and disrespect to care providers. In addition, several sub-themes were identified within these themes: Harsh and abusive language; Beating/slapping/pinching of the mother in labor; notably, Junior midwives also disrespected and abused women; Lack of privacy and confidentiality; Poor communication; No consent for maternity healthcare procedures; Lack of courtesy and poor interpersonal skills; and, negligence of care and woman’s needs.

**Conclusion:**

The actions of disrespect and abuse are alarming in practice and are associated with ignorance of fundamental human rights by both providers and recipients of services. Conducting workshops seems a useful approach to revealing disrespect and abuse deep-rooted in practice and provides an opportunity to rectify the problem with providers. A more extensive interventional study will be crucial to address the widespread actions of disrespect and abuse.

## Introduction

Globally, disrespectful, and abusive maternity healthcare is reported as violating women’s rights. The evidence depicts the negative consequences of disrespect and abuse of women seeking maternity healthcare services and poor quality of care [1–4]. This contradicts efforts directed toward the human rights agenda and universal health coverage.

Respectful maternity care (RMC) is a basic human right entitled to every childbearing woman, including the right to dignified and respectful care during pregnancy and childbirth [5]. Respectful care includes providing safe, evidence-based care that is provided in a supportive and friendly manner in a conducive environment with the involvement of women and their partners [6]. Evidence shows an association between respectful maternity care, improved quality of care, and utilization of maternity healthcare services [3, 6].

Disrespect and abuse vary in the types and extent of the mistreatment between countries [1, 7]. In Tanzania and other African countries, the common forms of disrespect and abuse include physical and verbal abuse, abandonment, soliciting of payment for service, retention in the facility for not being able to pay the bills, unconsented care, and the violation of privacy and confidentiality (of both physical and personal information) [1, 8–12]. A study to measure the prevalence of disrespect and abuse in Kenya revealed a rampant existence of this problem, including women who are left unattended, retained in the facility when they failed to settle bills, discriminated against, lack information and involvement in care provision and, their privacy and confidentiality violated [13, 14].

Disrespectful and abusive care is associated with women stopping seeking maternal health services from health facilities. In Tanzania, only 50% of childbirth in 2010 [15] and 62% in 2016 [16] utilized maternity healthcare services, and disrespect and abuse are highlighted among the reasons [17]. Women in rural Tanzania have declared that they would deliver at a health facility if maternity healthcare providers would use positive language and welcoming approaches [17].

Despite the growing anecdotal evidence in Tanzania, which sheds light on understanding disrespect and abuse, studies, and interventions to explore the root causes and solutions are limited. Our experience of working in the context and encountering several disrespectful and abusive cases in maternity services (e.g., use of harsh language, negligence of care, unconsented care, stereotyping and abandonment of patients) formed a basis for designing and conducting the study with healthcare providers, used as an avenue to explore potential problems and solutions.

### Aim

This study aimed to explore maternity healthcare provider’s perspectives on the disrespect and abuse experienced by women attending maternity services at health facilities in the Dar es Salaam region. Specifically, the study was designed to:

1. Analyze the current practices considering the maternity healthcare provider’s personal experience through critical reflections and self-assessment
2. Create awareness of standard guidelines for promoting respectful and quality Maternity services among healthcare providers.
3. Identify disrespect and abusive care in maternal healthcare

### Outcome

The benefits of this study were twofold: firstly, the study generated several themes around the public cry concerning the disrespectful and abusive care encountered by women seeking maternity services at healthcare facilities in Dar es Salaam. Secondly, the workshop fulfilled the requirement for the continuous professional development mandated by the Tanzania Nurses and Midwives Council to be undertaken by nurses and midwives before the renewal of their practicing license.

## Methods

This study used a descriptive qualitative design to explore maternity healthcare providers’ perspectives on the disrespect and abuse experienced by women attending maternity services at health facilities in the Dar es Salaam region. Qualitative research usually focuses on how and why enquiries, which allows a deeper understanding of the phenomena, situations, and encounters [18].

We conducted a series of three workshops of 20–30 participants each (30 from Ubungo and Temeke, 30 from Kigamboni and Kinondoni, and 20 from Ilala district); the workshop duration was three days,’ which was used to generate detailed data from participants. Workshops are increasingly applied where researchers wish to explore the challenges arising from complex phenomena and to identify solutions creatively and with stakeholders [19, 21] Various approaches are used to design workshops, including the use of cookbooks and guidelines, the use of conceptual format with pre-designed activities, and the use of open format [19]. In the present study, we used an open format workshop, which allowed participants and facilitators to negotiate and influence the workshop as unanticipated phenomena emerged. The design of the workshops was guided by the existing evidence, anecdotal data, and inspired by the authors’ experiences. Researchers believe that disrespectful and abusive care is sometimes hidden or accepted as a culture or a normal practice by both the provider and the consumer of maternal healthcare services. Therefore, various approaches were utilized to grasp details and nuances from front liners maternity healthcare providers.

### Setting

The workshop took place at the Aga khan University-school of nursing and midwifery (AKU-SoNaM) in the Dar es Salaam region, Tanzania. The AKU-SoNaM is a department of the larger Aga Khan University network situated in Tanzania, Kenya, and Uganda. The workshop involved maternity healthcare providers (Nurses, Midwives, and Clinical Officers) from health facilities in all five districts of the Dar es Salaam region (Ilala, Kinondoni, Temeke, Ubungo, and Kigamboni districts). The Ilala district, where AKU-SoNaM is located, has a population of 4,364,514 [20]. About 120 health facilities are located in this region: one (1) National hospital, three (3) super-specialty hospitals, three (3) public regional referral hospitals, eight (8)faith-based and private referral hospitals, five (5) district hospitals, fifty (50) health centres, and twenty-seven (27) dispensaries [21]. Dar es Salaam was selected based on its diversity, several health facilities (public and private), and vast catchment areas which represent urban and semi-urban settings. In addition, the low, middle, and high economic class population and many healthcare professionals reside in this region.

### Participants and recruitment

Eighty (80) participants were drawn from 30 public and private facilities, including dispensaries, health centres, and all hospitals in the Dar es Salaam region.

The office of the regional nursing officer coordinated the recruitment, and heads of facilities were approached via mobile phones and requested to identify providers with more than six months of experience working at antenatal clinics, labour wards, and postnatal clinics. Researchers view six months at a workplace to be long enough for someone to have exposure to or encounter cases of disrespect and abuse.

### Data collection

This study used different strategies to obtain in-depth information from healthcare providers during the workshop. First, the participants were asked to reflect on their practice individually. Second, they were asked to write anonymously about any incident that mirrors disrespect and abuse witnessed, experienced, or committed by themselves and stick this on the notice board. The participants were provided with descriptions to guide their reflections (Table 1).

**Table 1:** The guide for individual reflection of practice.

Reflect on your practice and others at your workplace or another health facility. What incident(s) do you consider mirrors disrespectful and abusive practice that you have witnessed being committed, experienced as a client or relative, or done to women/family while providing care/receiving healthcare? Anonymously summarise one incident and post it on the noticeboard. Please DO NOT write your name or any reference that may reveal the identity of the individual or the facility.

The short lectures on respectful maternity care and disrespect and abuse were conducted. This was followed by watching a video on “Why Mrs X. died” [22]. The RMC training material was developed by Aga Khan University using evidence-based materials, including the national and WHO guidelines and the White Ribbon Alliance Respectful Maternity Care Framework on RMC.

Subsequently, these sessions were followed by an interactive discussion with the larger group. Finally, participants were randomly organized into smaller groups and worked on a real-life scenario. This scenario (Table 2) constructed by one of the facilitators based on the case encountered in the health facility was used as a departure to a detailed discussion regarding real issues in the current practice. The group members nominated a chair who facilitated the discussions and a note taker who ensured that all the key points were captured. The discussions were also recorded using a digital recorder after verbal consent from the participants.

**Table 2:** Scenario.

An obstetrician approaches a mother in labor and tells her to lie down. With no warning, he performs a vaginal examination to evaluate her labor progress. As this examination was very painful, the woman requested the doctor to stop. He replies that the exam is almost over and completes the procedure despite her protests. Afterwards, a nurse-midwife tells a mother in labor that her baby is in distress and must be born quickly. The midwife informed the mother that she needs a painful and expensive procedure, or her baby will die. When she asks if there are other options, the nurse-midwife yells at her and says she is wasting time and needs to deliver immediately. The woman agrees to the procedure. Fortunately, a woman gave birth to a baby girl in good condition. The midwives called the husband and gave the bed sheets stained with birth products (meconium, blood etc) for washing. This man took the cloths without questioning for washing at a nearby water tap. As he started washing, a security guard who sat close to the tap shouted at him that he was using water unwisely. Another midwife, who was walking towards the maternity block while this guard was shouting also added “*ndiyo zao hizo*” meaning that “this is how relatives misuse water”. 1. With a reflection to daily practices and experiences at your health facility, discuss this scenario from your point of view. 2. Discuss respectful, disrespect and abusive care concepts as related to care of the mother and relatives at your context. 3. What are the common disrespect and abuse done knowingly or unknowingly or witnessed/experienced any time during the childbirth continuum (pregnancy, birth and postnatal)?

### Data analysis

Data drawn from two data sets: individual responses and notes from group discussions were analysed following six steps as described by Braun and Clark [23]. The Nvivo version 12 computer software was used to aid the data organization and analysis. The first author listened to the recorded group discussions assimilating with the transcripts produced from the discussion and read all the transcripts repeatedly to familiarize herself with the data, understand the meaning arising, to exclude any misunderstanding of the participant’s views. The descriptive codes, which were closely related to the content of the data, were generated. All authors reviewed the coded data, and similar codes which formed the meaningful pattern of data were grouped to formulate sub-theme and themes. These were checked against codes and sometimes with the entire data set to ensure that they were relevant. The overlaps and repetitions were observed and adjusted based on all authors’ agreement.

### Ethics

The Aga Khan University ethics review committee approved the study and issued a certificate with reference number: Ref: IED/2018/010/fb/016. The workshop course outlines were accredited and approved by Tanzania Nurses and Midwives Council (TNMC) before the onset of the workshop. The TNMC requires all nurses and midwives to undergo continuous professional development courses in accredited institutions before renewing their practicing licenses. Therefore the workshop conducted in this study was designed to fulfill the requirement for continuous professional development for nurses and midwives. The Dar es Salaam Regional Administrative officer and health facilities management granted permission to allow maternity healthcare providers to participate in the workshop. In addition, the researchers explained the process and purpose of the study to participants and asked for their verbal consent before the data collection. Researchers did not anticipate any serious risk during the running of the workshop and data collection. However, as the methodology involved disclosure of personal practices of disrespect and abuse, precautions were taken to ensure personal identity is not disclosed. Participants were assured of the confidentiality of the information shared during the workshops and informed of their voluntary participation. No names or any individual’s identity appeared on notes where individual or group responses about disrespect and abuse were written. Participants were informed that they could withdraw at any time when wanted to do so and were not forced to share any information.

## Results

Eighty (80) healthcare providers from various health facilities, including dispensaries (*n* = 25), health centres (*n* = 2), and hospitals (*n* = 3) located in Dar es Salaam, attended the workshop. The age of participants ranged from 25 to 45 years. All had more than two (2) years of working experience in maternity care services. Most of the participants were female.

Four main themes were identified from the data: *Physical and verbal abuse*; *Lack of professional ethics and integrity*; a *Vulnerable working environment;* and *disrespectful women and relatives*, see details on Table 3.

**Table 3:** Presents the major themes and sub-themes.

Main theme	Sub-theme
Physical and verbal abuse	• Harsh and abusive language
	• Beating/slapping/pinching of the mother in labor
	• Junior midwives also disrespected and abused
Poor adherence to professional ethics and integrity	• Lack of privacy and confidentiality
	• Poor communication
	• No consent for maternity care
	• Lack of courtesy and poor interpersonal skills
	• Negligence of care and women’s needs
Vulnerable working environment	• Unfulfilled policies
	• Space constraint
	• Inadequate resources
	• Shortage of competent staff
	• Inadequate compensation and recognition
	• Unsupportive supervisors
Abuse and disrespect to care providers	

### Physical and verbal abuse

Physical and verbal abuse was described as a more common practice among other types featuring various forms: harsh or abusive language, yelling, stereotyping, and/or beating women, and pinching of mothers, particularly during labour pain and delivery. For example, while describing how the slapping was common, a participant proudly explained that:

*Myself and a few of my colleagues at our workplace are nicknamed commandos because when we are on shift*, *we never end up with birth asphyxia*. *We are very tough with women during labour*. *If women fail to collaborate*, *they could be slapped*, *pressure applied to rescue her life and that of a child*.”

Another participant explains below:

*She said to me (senior Midwife) that if you’re not harsh*, *the woman will not push out the baby*, *and you will cause a delayed delivery and feotal complications or death*.

Paradoxically, women consider the disrespect and abuse as a help and a way of rescuing the baby while to the midwife, is an act of bravery and life-saving for the woman and the baby.

*“Two weeks after the discharge from the hospital*, *the woman who I had slapped during birth*, *came back to thank me for saving her baby’s life and brought me a pair of “Kitenge” (respected cultural cloth material) as a token of appreciation*.”

More comments were provided:

*Some health care providers use “Wanatumia maneno makali sana” meaning harsh and abusive language*, *such as “nilikutuma” (meaning I did not ask you to conceive) (From individual reflection]*. *You are disturbing me (Individual reflection)*. *Another example is the bad use of language like “your baby will die if you don’t listen to me”(Group discussion* 2)

This study revealed that senior midwives often abuse and disrespect junior colleagues the way they do to clients.

*“A senior midwife almost beat me because I supported a mother to go through labor politely*. *She (senior midwife) yelled and slapped the mother and also attempted to slap me*, *but I managed to escape*.”

Interestingly, participants queried common questions they ask women as constituting disrespect and abuse. Some of the statements considered inappropriate are

*Why don’t you have money*? *Where is your partner*? *Why did you come late*? *Do you want to kill your baby*?

However, most participants agree they should change this practice and free women from questioning as they may have no answers when they seek care.

### Poor adherence to professional ethics and integrity

Study findings indicate the existence of practices that violate professional ethics and integrity. Anonymously, the participants confessed that they often provide care without consideration of the client’s privacy and confidentiality, not asking for consent and inadequate communication among providers and with women/relatives about care and decisions made during childbirth. Some of the common responses from the participants when asked to report the kind of abuse and disrespect include:

*Poor communication among health providers and clients*, *Poor customer care*, *failure to provide quality care*, *Use of poor/harsh language*, *provision of inadequate information about their care*, *Procedures are sometimes performed without the patient’s consent; even when consent is obtained*, *the explanation given to clients is incomplete and Use of poor language/harsh words and poor history takin*g.

### Vulnerable working environment

The most common issues highlighted in the theme are lack of working equipment and supplies, space constraints, confusing process, high workload, lack of commitment from leadership, unclear and unfulfilled policies (e.g., free treatment for pregnant women and under five), and shortage of qualified staff. Some of the comments made by the participants are:

*We are few with many patients*, *and sometimes*, *we run out of supplies*, *e*.*g*., *Pitocin and gloves*. *In addition*, *the space is small and congested impossible to provide privacy (Group discussion -1*,*2*,*3*, *4 and 5*).

*No recognition of what we do*, *and worse*, *if something goes wrong*, *you are blamed without getting the opportunity for explanations (individual reflections*).

*When women and their families come to the health facility*, *they expect to get free treatment*, *and if they don’t*, *we are in trouble we are accused of theft (Group discussion 4*).

### Abuse and disrespect to care providers

This study revealed existence of abuse toward health care providers from women and their relatives commonly verbal and sometimes physical. In addition, participants reported unsupportive supervisors or senior staff and politicians. The examples given were harsh language, unfair judgement and treatment, and overwork. Typical example comment from participants | “*what about us when dealing with disrespectful women and their relatives*?*” (Group discussion 2) ……women and family expect to get free treatment*, *and if they don’t*, *we are in trouble we are accused of theft (Group discussion 4)when clients report anything negative most of the time we are unfairly judged by supervisors*, *politicians and community before they listen to our side of the story*. *(group 1) were always short-staffed and hence overwhelmed with work*.

## Discussion

The present study explores maternity healthcare providers’ perspectives on the disrespect and abuse experienced by women attending maternity services at health facilities in the Dar es Salaam region. The key findings reveal the apparent existence of disrespectful and abusive care in maternity services. As a result, four main themes were identified from the data: *Physical and verbal abuse*; *Lack of professional ethics and integrity*; a *Vulnerable working environment;* and *Abuse and disrespect to care*, *providers*.

These findings correspond with the evidence from various studies which assert the prevalence of disrespect and abuse of facility-based maternal healthcare services, hindering the achievement of positive maternal health outcomes [10, 12, 24]. Surprisingly, the findings in the current study indicate that some healthcare providers associate disrespect and abuse with positive birth outcomes, such as beating/pinching/and threatening women in the second stage of labor to help push the baby out. Unfortunately, the notion is accepted by some women as help believing they would not have made it if the midwife did not intervene. From these findings, the researchers do not undermine the fact that providers may lack awareness of women’s rights in the services they provide [25], and that sometimes they disrespect and abuse them unknowingly. On the other hand, it is also apparent that most women and their families do not understand their rights in reproductive health services. Therefore, we engaged with participants to clarify areas that felt unclear as they emerged. For instance, we discussed basic human rights in reproductive health and explored different strategies for dealing with various situations. As a result, most participants spontaneously and, as a shock, learned what they do as a normal practice violates women’s rights. Notably, the incidents of lack of awareness of the patient’s rights have been reported in other studies [22, 25].

Similar to this study, several global studies have widely reported physical and verbal abuse [4, 7, 13, 26]. For instance, a recent study conducted in India and Iraq reported that shouting, scolding, and yelling is the most common form of abuse women experience, which deters women from utilizing maternity services [26, 27]. In addition, the primary concern raised by women in Iraq was the lack of privacy. At the same time, physical and verbal abuse was pointed out in the present study as a major problem.

Unlike the previous studies, this study revealed that senior midwives often abuse and disrespect junior colleagues the same way they would clients. Yelling and slapping were commonly happening to these professionals during their early days in practice. Participants believe that junior maternity providers who encounter disrespect and abuse are likely to learn and inherit the practice from their mentors. Disrespectful care and abuse may become a generational problem if not addressed appropriately. The implication stated here resonates with the pedagogy of the oppressed theory [28]; when a person is oppressed by their superior is likely to behave the same if the opportunity arises.

Participants described the practices that reflect a lack of professional ethics and integrity, including lack of confidentiality and privacy, poor provider-client communication, and none consented care/procedures. In addition, to a lack of courtesy, poor interpersonal skills, negligence of care and women’s needs, and delayed care. Several studies globally have reported similar findings to the present study [4, 29, 30]. Accordingly, participants believe these actions are committed knowingly since professional ethics and integrity is among the core courses in all healthcare professions, which is also emphasized in the professional code of conduct. Evidence from global perspectives agrees with this study’s findings in most cases described here; despite receiving professional training, many incidents of unprofessional care are reported [2, 3, 31]. For example, a study in Sweden reported that women felt excluded in decisions made about measures taken by staff during childbirth and were given unclear and incomplete information. In several cases, they received no information at all [4]. A study in Ethiopia also reported that women often receive non-confidential, unconsented, and delayed care [29].

Participants in the present study feel that disrespect and abuse are complex and may be caused by an individual provider who lacks professional ethics and integrity. However, several systemic problems exaggerate the problem. They cited lack of resources and poor leadership as contributing to the bridging of professional ethics and integrity, e.g., lack of screening curtains and private rooms affect privacy and confidentiality during care. Studies in other areas have reported similar findings [9, 17, 32]. These findings imply that environmental factors are essential components that may influence the client’s care.

It will be interesting to find out what factors contribute to the lack of professionalism and what can be done to overcome the situation from the health care professional’s perspective.

Concerning the work environment, this study’s findings revealed dissatisfaction with the work environment among healthcare providers. Participants felt that their working environment and the system-related issues directly or indirectly expose them to the risk of committing disrespectful and abusive care. The main areas of concern were; lack of working equipment and supplies, space constraints, unclear processes, high workload, lack of commitment from leadership, vague and unfulfilled policies (e.g., free treatment for pregnant women and under-five children), and shortage of qualified staff. These findings are similar to those in several studies from within and in other countries [17, 26, 30, 32, 33]. For example, a study in Tanzania reported a lack of partitions between beds and that women shared beds [32]. It implies that even if one wanted to provide respectful care in such a situation, it would be hard to do so. As a result, women/families will continue complaining of poor care. In contrast, the health care providers remain frustrated and unhappy, which may inversely affect the care and health outcomes for the mother and the child.

On the contrary, findings in the current study revealed that although the emphasis is on addressing disrespect and abuse against women, healthcare providers are also disrespected and abused Participants wanted to know how and who would address disrespect and abuse from women and their families, asking, “*what about disrespectful women and relatives*?” Previous studies have reported clients abusing healthcare providers verbally and physically due to emotional reactions and stress [34]. The participants also cited unsupportive systems, and judgmental communities. A lengthy discussion ensued following this question, insisting that some healthcare providers suffer silently from disrespect and abuse experienced from women and their relatives, supervisors, and the challenging work environment. Furthermore, they think disrespected, and abused professionals will likely disrespect and abuse innocent clients. Finally, participants expressed their enthusiasm for understanding their rights which would balance with the discussion around disrespect and abuse of women at maternity services.

Violence against healthcare providers has been relatively reported worldwide however, there is a paucity of literature on women and relatives’ disrespect and abuse of maternity healthcare providers in Tanzania. However, a study in Tanzania reported that health providers might act disrespectfully due to the existing inequality, where they hold authority over women, though they are the victims of continuous health system challenges and poor working conditions [17].

## Limitation

The researchers acknowledge that this study uses nurses, midwives, and clinical officers as prototype of providers to examine their perspectives of disrespect and abuse in maternal newborn, and child health care in Dar es Salaam. However, maternal health care is provided by a multidisciplinary team of providers; hence participants’ discernments of disrespect and abuse may mirror such communications. Conducting a similar study extending beyond nurses, midwives, and clinical officers; and in different settings than urban and semi-urban contexts may influence a significant positive change in the problem. Notwithstanding the limitation, the researchers believe that the evidence generated from this study will form a foundation for lager intervention study focusing on addressing the chronic problem of disrespect and abuse.

## Conclusion

Act of disrespect and abuse is alarming in practice; it is associated with ignorance of fundamental human rights by providers and recipients of services. Conducting workshops can be useful in revealing disrespect and abuse deep-rooted in practice from provider’s perspectives and provides an opportunity to rectify the problem with providers. The findings of this study suggest the crucial need of more extensive interventional study to address the widespread actions of disrespect and abuse.
